# Flexible Neural Probes with Electrochemical Modified Microelectrodes for Artifact-Free Optogenetic Applications

**DOI:** 10.3390/ijms222111528

**Published:** 2021-10-26

**Authors:** Bangbang Guo, Ye Fan, Minghao Wang, Yuhua Cheng, Bowen Ji, Ying Chen, Gaofeng Wang

**Affiliations:** 1 Wenzhou Institute of Hangzhou Dianzi University, Wenzhou 325038, China; bbguo@hdu.edu.cn (B.G.); 16041701@hdu.edu.cn (Y.F.); chengyh@hdu.edu.cn (Y.C.); 2 MOE Engineering Research Center of Smart Microsensors and Microsystems, School of Electronics and Information, Hangzhou Dianzi University, Hangzhou 310018, China; 3 The Unmanned System Research Institute, Northwestern Polytechnical University, Xi’an 710060, China; bwji@nwpu.edu.cn; 4 The Institute of Flexible Electronics Technology of THU, Jiaxing 314000, China; chenying@ifet-tsinghua.org

**Keywords:** optogenetics, photoelectric artifact, Pt-Black/PEDOT-GO, neural recording, optical stimulation

## Abstract

With the rapid increase in the use of optogenetics to investigate nervous systems, there is high demand for neural interfaces that can simultaneously perform optical stimulation and electrophysiological recording. However, high-magnitude stimulation artifacts have prevented experiments from being conducted at a desirably high temporal resolution. Here, a flexible polyimide-based neural probe with polyethylene glycol (PEG) packaged optical fiber and Pt-Black/PEDOT-GO (graphene oxide doped poly(3,4-ethylene-dioxythiophene)) modified microelectrodes was developed to reduce the stimulation artifacts that are induced by photoelectrochemical (PEC) and photovoltaic (PV) effects. The advantages of this design include quick and accurate implantation and high-resolution recording capacities. Firstly, electrochemical performance of the modified microelectrodes is significantly improved due to the large specific surface area of the GO layer. Secondly, good mechanical and electrochemical stability of the modified microelectrodes is obtained by using Pt-Black as bonding layer. Lastly, bench noise recordings revealed that PEC noise amplitude of the modified neural probes could be reduced to less than 50 µV and no PV noise was detected when compared to silicon-based neural probes. The results indicate that this device is a promising optogenetic tool for studying local neural circuits.

## 1. Introduction

The emergence of optogenetic techniques in neuroscience has provided many new methods to study the effects of specific subpopulations of neurons on behavior. Based on this technology, a widespread approach is to combine optical fiber with electrical recording microelectrodes [1,2,3,4]. However, these approaches are difficult to implement for several reasons. On one hand, neural probes with fibers are inflexible for optical stimulation because of the need for external light sources. On the other hand, the fiber-tangle restricts the free movement of animals and prevents high-density integration [5,6,7,8,9,10,11,12,13].

To overcome these problems, active neural probes that integrate an LED/LD (all the abbreviations in the paper are explained in Table 1) directly onto a neural probe have been developed to provide high stimulus resolution [14,15,16,17,18,19,20,21,22,23,24]. However, the intrinsic disadvantages limit the further applications of these active neural probes. Firstly, the integrated LED/LD on probe generates heat, causing thermal damage to brain tissue during optical stimulation. Secondly, the high-density recording channels near the LED/LD are susceptible to parasitic capacitors, generating stimulation-locked electromagnetic interference (EMI) noise. Thirdly, the metal microelectrodes exposed to light radiation are susceptible to photoelectric effects, generating stimulation-locked photoelectric artifacts [25,26,27,28,29,30]. Finally, the efficiency of direct optical coupling between LED/LDs and optical fiber/waveguides is very low, which results in insufficient output optical power and increases the amplitude of the artifacts [31,32,33].

In recent years, many works in the literature have reported on methods for mitigation of thermal damage, coupling loss, and EMI noise in active neural probes [34,35,36]. K. Kampasi et al. used a grounded brass shield to limit the amplitude of EMI noise to less than 100 μV under an optical output power of 450 μW [21]. They also demonstrated successful device implementation by achieving efficient coupling between an LD and a dielectric optical waveguide mixer via a gradient-index (GRIN) lens. The use of GRIN lenses attains several design features, including high optical coupling and thermal isolation between LD and waveguide. K. Kim et al. presented a multi-metal-layer structure with a shielding layer that effectively suppresses the EMI noise of stimulation signals. In addition, a heavily boron-doped silicon substrate was used to suppress PV artifacts caused by LED illumination [23]. However, the use of heavily doped silicon substrate still cannot eliminate the photoelectric noise completely due to the presence of PEC noise. Meanwhile, the mechanical mismatch between the rigid probe and neural tissue may affect the long-term stability of the neural interface.

Recently, flexible polymer-based neural probes have been reported [37,38]. Advantages of the flexible polymer-based neural probes over the rigid silicon-based neural probes include low elastic modulus, good biocompatibility, and low mechanical mismatch. However, the fast and precise implantation of these flexible neural probes in brain can be a problem due to their small buckling force. For this, W. Ling et al. developed a neural probe that was surface-coated with polyvinyl alcohol (PVA) or polyethylene glycol (PEG) to enhance the rigidness of the probe to avoid its bending. Both PVA and PEG could dissolve in the cerebrospinal fluid after implantation to restore the flexibility of the probe. In addition, several other biodegradable materials have been proposed for the hardening of flexible probes, such as maltose and silk protein [39,40]. However, these works have not yet integrated the function of optical stimulation, and the study of stimulation artifacts is relatively lacking.

Since the amplitude of PEC noise generated by a metal microelectrode can exceed 100 µV when exposed to light illumination [41], transparent conductors with a band gap of more than 3.26 eV, such as counterion doped PEDOT, tin-doped indium-oxide, or graphene, are often proposed as interface materials for neural probes [42]. J. Park et al. proposed a microelectrode array based on PEDOT:PSS/graphene, whose PEC noise is less than 40 µV under blue light irradiation of 30 mW/mm^2^ [43]. L. Wang et al. has demonstrated that Pt-Black/PSS modification can decrease the PV and PEC noise of silicon-based probes to less than 250 µV under blue light irradiation of 38.2 mW/mm^2^ [44]. However, the PV noise cannot be eliminated completely due to the use of silicon substrate. Moreover, the PV noise and PEC noise of a silicon-based probe are always mixed together, which makes it difficult to analyze the mechanism of electrochemical modification to reduce the photoelectric artifact.

This work introduces a polyimide (PI)-based probe with electrochemical modified microelectrodes for artifact-free optogenetics. Firstly, a polyimide (PI)-based probe was fabricated by micromachining technology and integrated with PEG packaged optical fiber to measure the optical stimulation noises. Secondly, the photoelectric noise generated by the PV effect was eliminated by using polyimide as substrate instead of silicon. In addition, the Pt-Black (PBK) and PEDOT-GO (PGO) were deposited on the PI-based probe successively to mitigate background noise and PEC noise. The electrochemical and signal recording performance were studied and compared to the reported work in Table 2, and the results show that this device is promising in high-resolution and artifact-free optogenetics.

## 2. Results

### 2.1. Morphology Characterization

**Figure 1:**
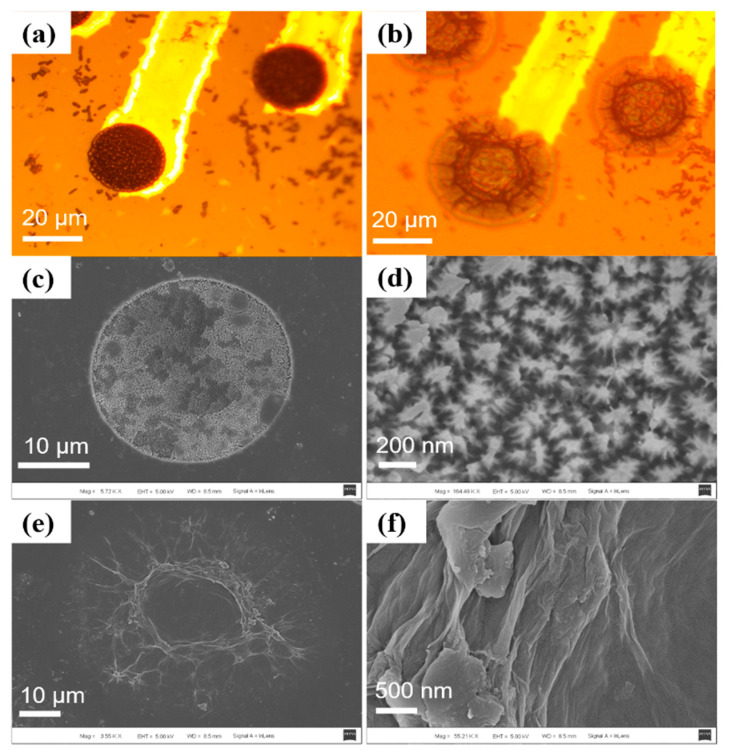
Micrograph of (**a**) PBK modified microelectrode and (**b**) PBK/PGO modified microelectrode. SEM pictures of (**c**,**d**) PBK modified microelectrode and (**e**,**f**) PBK/PGO modified microelectrode with large effective surface area.

The morphology of the modified microelectrodes was observed by optical microscopy and scanning electron microscopy (SEM). As can be seen in Figure 1a, the optical micrograph shows PBK deposited on the bare gold microelectrodes tightly and uniformly without cracks. Furthermore, the SEM pictures in Figure 1c,d show that the PBK has many cauliflower-shape nanoparticles with dimensions of several hundred nanometers. These nanoparticles gave the PBK a large specific surface area, which could enhance the binding force with PGO. An optical micrograph of the PBK/PGO modified microelectrodes is shown in Figure 1b. As can be seen, the PGO was deposited on PBK evenly and tightly without delamination. Furthermore, the SEM pictures in Figure 1e,f show the electrodeposited PBK/PGO has a folded microstructure with large specific surface area, which helps to increase double layer capacitance.

### 2.2. Electrochemical Characterization

**Figure 2:**
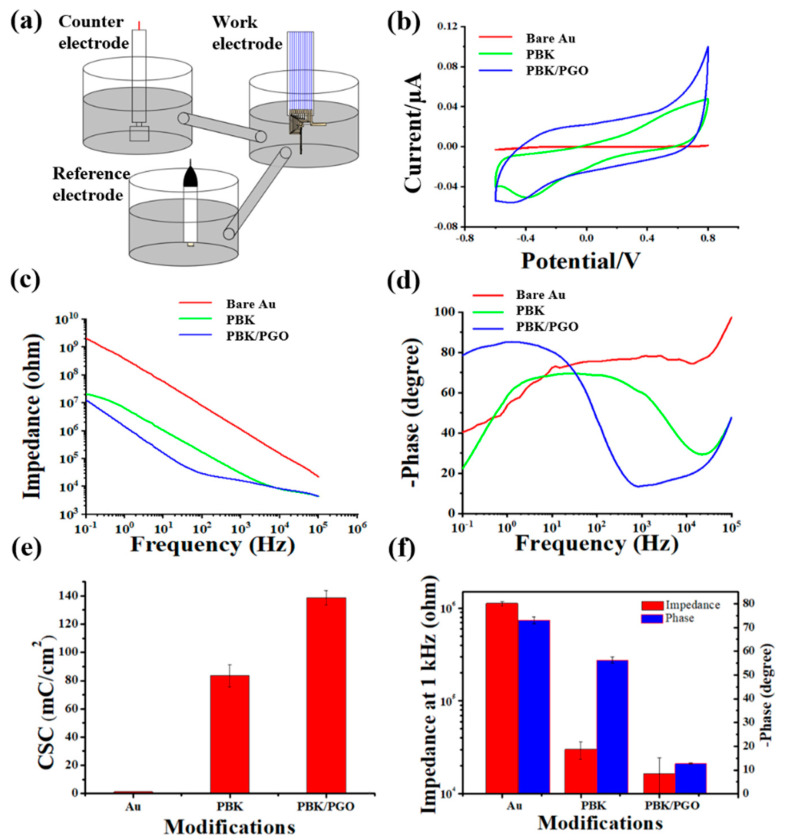
(**a**) Three-electrode system, (**b**) averaged cyclic voltammetry curves, (**c**) electrochemical impedance spectra, and (**d**) phase curves of unmodified, PBK modified, and PBK/PGO modified microelectrodes (*n* = 3). (**e**) Histogram of CSC of microelectrode before and after modification. (**f**) Histogram of impedance and phase at 1 kHz of microelectrode before and after modification.

As illustrated in Figure 2a, the three-electrode system was composed of a work electrode, a saturated calomel electrode (SCE), and a Pt foil counter electrode. For electrochemical characterization, cyclic voltammograms (CV) were scanned between −0.6 V to 0.8 V at 0.1 V/s, and electrochemical impedance spectra (EIS) were measured from 10^−1^ Hz to 10^5^ Hz with 10 mV input voltage in PBS. The scanning range of −0.6V to 0.8 V was chosen because higher voltage amplitudes may cause damage to biological tissues by electrolysis of water, and −0.6V to 0.8 V is the range of water windows where water is not electrolyzed. The CV and EIS data from three microelectrode sites of the probe were measured and calculated with averaging for the unmodified, PBK-modified, and PBK/PGO-modified microelectrodes. As shown in Figure 2b, the enclosed area of the CV curves represents the charge storage capacity (CSC) of the microelectrodes with different modifications. After calculation, the CSC of the bare Au microelectrodes was significantly increased, from 1.3 ± 0.1 mC/cm^2^ to 83.4 ± 8.0 mC/cm^2^ and 138.7 ± 5.1 mC/cm^2^ after PBK and PBK/PGO modification, as depicted in Figure 2e. The reason was that PBK and PGO are both pseudocapacitive materials with large specific surface areas, so they both have large pseudocapacitance and double-layer capacitance that can increase the CSC of the modified microelectrodes dramatically. The CSC of PBK/PGO modified microelectrodes was much larger than that of the PBK modified ones, which was because the folded multilayer PGO structure had a larger specific surface area. From Figure 2c,d, the impedance and phase delay both decreased significantly at 1 kHz after modification. After the average calculation, the impedance and phase delay at 1 kHz, respectively, decreased from 1124.0 ± 65.0 kΩ and 78.0 ± 1.5° to 30.0 ± 6.5 kΩ and 60.1 ± 1.4° after PBK deposition, and then to 16.5 ± 7.7 kΩ and 13.6 ± 0.3° after PBK/PGO deposition, as depicted in Figure 2f. It can be seen that PBK made a great contribution to the reduction of impedance, while PGO made a great contribution to the reduction of phase delay. The lower impedance means higher signal-to-noise ratio (SNR), while the smaller phase delay means less waveform distortion. Therefore, PBK/PGO modification could improve the SNR and decrease signal distortion simultaneously for neural signal recordings.

### 2.3. Stability Tests

**Figure 3:**
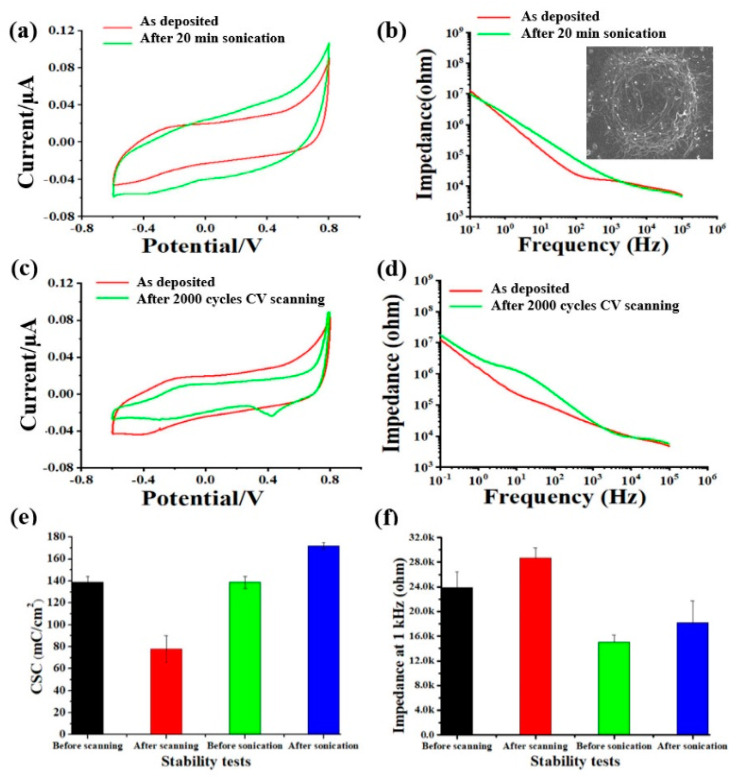
(**a**) Averaged CV plots and (**b**) impedance spectra of the PBK/PGO modified microelectrode before and after 20 min of ultrasonication (*n* = 3). The inset shows SEM picture of one PBK/PGO modified microelectrode after 20 min of ultrasonication. Corresponding (**c**) CV plots and (**d**) impedance spectra before and after 2000 cycles of CV scanning (*n* = 3). (**e**) Histogram of (**e**) CSC and (**f**) impedance at 1 kHz of PBK/PGO modified microelectrode before and after stability tests.

The mechanical stability of PBK/PGO was measured by applying ultrasonic agitation and CV scanning on the modified microelectrode. Firstly, the PBK/PGO modified microelectrode was immersed into PBS, where an ultrasonic agitation was applied with a power of 240 W to simulate the micromotion of brain tissue. After 20 min of ultrasonic agitation, the probe was removed from the PBS to measure the CV and EIS. As shown in Figure 3a,b,e,f, the averaged CSC value increased by 24.1%, and the averaged impedance at 1 kHz increased by 20.8% after ultrasonic agitation (*n* = 3). The corresponding SEM pictures of one modified microelectrode after sonication in Figure 3b reveal that the PBK/PGO was stable without delamination during sonication. The CV was scanned from −0.6 V to 0.8 V with a scanning rate of 1 V/s to study the electrochemical stability of PBK/PGO. After 2000 cycles of scanning, CV and EIS were measured as depicted in Figure 3c,d. As shown in Figure 3e,f, the calculated CSC exhibited a 43.8% decrement, and the impedance at 1 kHz showed a 20.1% increment after CV scanning (*n* = 3). Obviously, the CSC of the modified microelectrode decreased significantly after CV scanning, which was because the ultrasonic power used in the test was much greater than that used in the previous works. The changes in CSC and impedance after ultrasonic agitation and CV scanning demonstrate that the deposited PBK/PGO on microelectrode has adequate mechanical and electrochemical stability.

### 2.4. Equivalent Circuit Analysis

**Figure 4:**
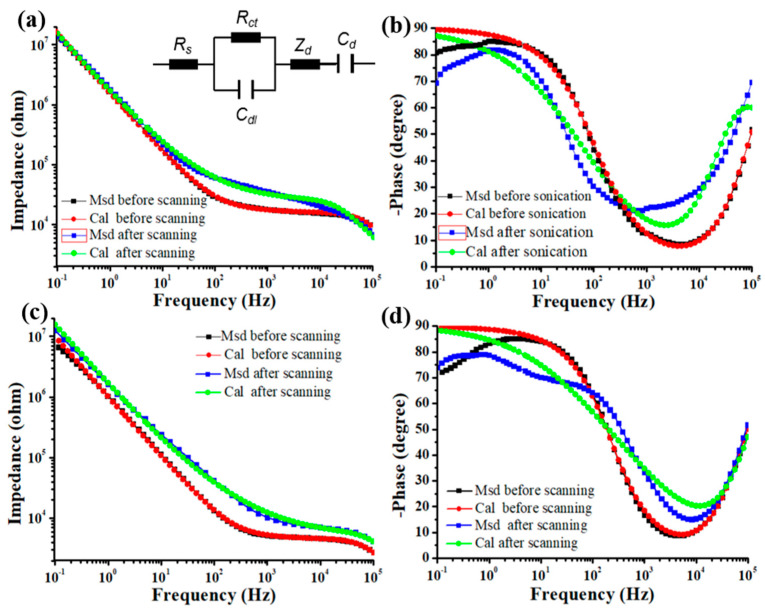
The measured and calculated impedance (**a**) and phase (**b**) spectra before and after 20 min sonication. The inset shows the fitting equivalent circuit. The measured and calculated impedance (**c**) and phase (**d**) spectra before and after 2000 cycles of CV scanning.

For PBK/PGO modified microelectrodes, the equivalent circuit model is illustrated in inset of Figure 4a, in which the components *R_s_*, *C_dl_*, *R_ct_*, *Z_D_*, and *C_d_* represent solution resistance, double layer capacitance, charge transfer resistance, bounded Warburg element, and bulk capacitance, respectively. The bounded Warburg element *Z_D_* can be further described as follows:

(1) $$Z_{D}=Y_{0}^{-1}{\left(j\omega \right)}^{-1/2}coth[B\left(j\omega {)}^{1/2}\right]$$

where *Y*_0_ and *B* are fitting parameters, *j* = (−1)^1/2^, and *ω* = angular frequency = 2πf. Based on the equivalent circuit model, the fitted component values of PBK/PGO modified microelectrodes before and after stability tests are illustrated in Table 3. The small chi-square test *χ*^2^ indicated that the equivalent circuit model provided fairly good fitting to measured data. According to the fitted curves in Figure 4a,b, the values of *R_ct_*, *Z_D_*, *C_dl_*, and *C_d_* all increased after sonication, which indicates that the charge transfer process and the electrochemical diffusion process slowed down, while the charge quantity distributed on the electrode-electrolyte interface and the electro-active surface area increased. The increase in *C_dl_* and *C_d_* may be the main reason for the increase in the CSC value shown in Figure 3a. According to the fitted curves in Figure 4c,d, the values of *R_ct_* and *Z_D_* both increased after CV scanning, which indicates the charge transfer process and the electrochemical diffusion process both slowed down. Meanwhile, the values of *C_dl_* and *C_d_* both decreased after CV scanning, which means the charge quantity distributed on the electrode-electrolyte interface and the electro-active surface area decreased. The fitted results reveal the reasons for the CSC and impedance variations in Figure 3c,d.

### 2.5. Mechanical and Optical Tests

**Figure 5:**
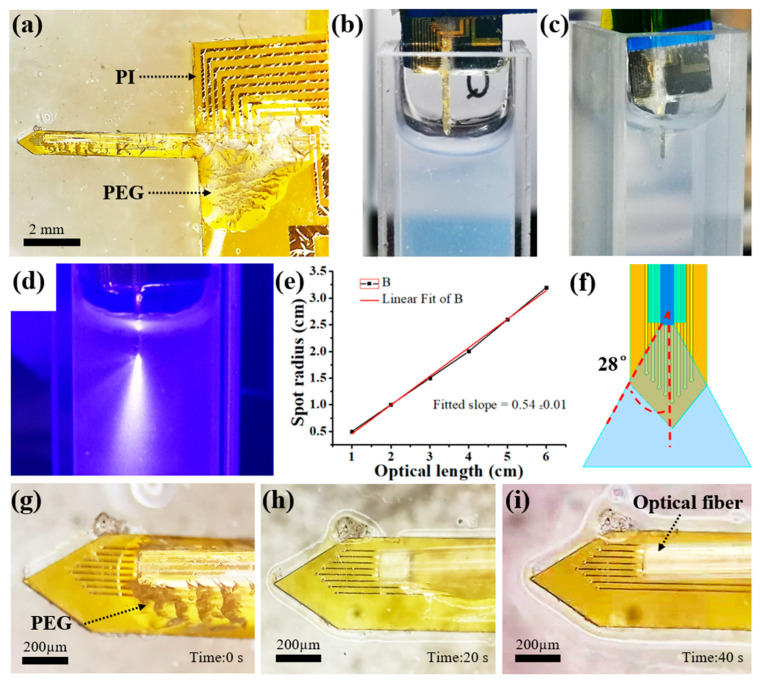
(**a**) Optical micrographs of a PI-based neural probe with PEG-packaged optical fiber. Optical micrographs of the PEG-packaged probe (**b**) before and (**c**) after implantation in agar gel. (**d**) Light tracer of the laser beam in agar gel. (**e**) The measured and fitted spot radius R at different distances L. (**f**) The laser beam model of a fiber on the PI probe. Optical micrographs of the PEG on the tip of optical fiber (**g**) before and after being dissolved in PBS for (**h**) 20 s and (**i**) 40 s.

Figure 5a shows a PI-based neural probe with PEG-packaged optical fiber. Obviously, the optical fiber could be fixed to the flexible PI probe tightly with the solidified PEG. In order to verify the mechanical properties of PEG encapsulated probe, a simulated implantation experiment was carried out using 0.8% concentration agar gel. Figure 5b,c depict the trajectory of the probe before and after implantation in the agar gel. The results showed the packaged probe could inserted into the agar gel without bending. Then, the divergence angle of the laser beam was observed and measured by the light tracer method and light intensity distribution measurement method, respectively. As shown in Figure 5d, the laser beam took on the shape of a light cone after the neural probe was inserted into the agar gel. To measure the divergence angle, a laser beam was projected vertically onto a piece of white paper in a dark room. Then, the divergence angle of the laser beam could be calculated by measuring the distance L of the optical fiber from the paper and the radius R of the spot on the paper. The spot radius R was defined as the distance between the spot center and the position where the light intensity was attenuated to 1/e^2^ of the central light intensity. Finally, the divergence angle θ can be calculated as:

(2) $$\theta =\arctan\frac{2R}{L}$$

In order to improve the measurement accuracy, the spot radius R at different distances L was measured and fitted as shown in Figure 5e. After calculation, the θ/2 was equal to 28°. The laser beam model of a fiber on the PI probe is illustrated in Figure 5f. Obviously, the divergent laser beam was sufficient to illuminate all the microelectrode sites when the distance L from the optical fiber to the microelectrode was about 200 µm. To test the degradation rate of PEG in vivo, the encapsulated neural probe was inserted into PBS solution to simulate the implantation process in tissue fluid. Figure 5g–i show the optical micrographs of the PEG on the tip of optical fiber before and after immersion in PBS solution. As can be seen, the PEG dissolved quickly after soaking in PBS for 40 s. Therefore, this design ensures that the neural probe recovers flexibility immediately after implantation, thus reducing the inflammatory response of the tissue.

### 2.6. Bench Noise Recordings

**Figure 6:**
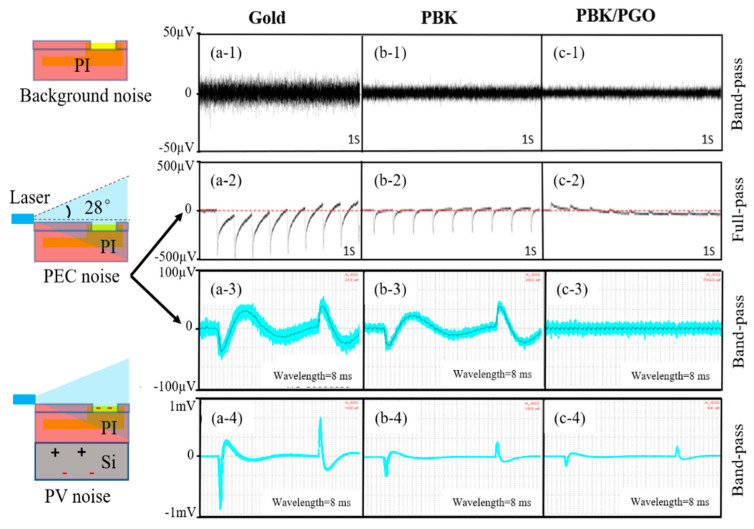
Background noise recorded from (**a-1**) gold microelectrode, (**b-1**) PBK modified microelectrode, and (**c-1**) PBK/PGO modified microelectrode under laser pulse stimulation (0.2 A, 5 ms, 10 Hz) on bench. Recorded (**a-2**,**b-2**,**c-2**) full-pass and (**a-3**,**b-3**,**c-3**) band-pass PEC noise of PI-based probe under laser pulse stimulation (0.2 A, 5 ms, 10 Hz, *n* = 100). (**a-4**,**b-4**,**c-4**) Recorded photoelectric artifacts of silicon-based probe under laser pulse stimulation (0.1 A, 5 ms, 10 Hz, *n* = 100).

The recorded waveforms of background noise, PEC noise, and PV noise are presented in Figure 6. As shown in Figure 6(a-1,b-1,c-1), the averaged amplitude of the background noise decreased from 9.0 ± 1.4 μV to 7.0 ± 0.9 μV after PBK modification, and then to 5.0 ± 1.6 μV after PBK/PGO modification, suggesting that background noise decreases with the decline in electrode impedance. The photoelectric artifact of the probe was mainly composed of two parts: one originated from the PV effect of the silicon substrate, and another originated from the PEC effect of the microelectrode. Due to the lack of silicon substrate required for the PV effect, the photoelectric artifact of the PI-based probe could have originated only from the PEC effect. As can be seen in Figure 6(a-2,b-2,c-2), the all-pass PEC artifacts of gold and PBK modified microelectrodes from the PI-based probe both had V-shaped waveforms when the light pulses were turning on. However, the PEC artifacts of PBK/PGO modified microelectrodes had an “inverted-v” (or “^”)-shaped waveform. The polarity of the two waveforms was opposite because the PEC of gold/PBK (metal) led to the accumulation of electrons, whereas the PEC of PGO (conductive polymer) led to the accumulation of holes. A similar “^”-shaped waveform originated from the PEC effect of PEDOT has been described in reference [43]. After calculation, the average amplitude of the all-pass PEC noise dropped from 316.0 ± 69.9 µV for unmodified microelectrode to 215.0 ± 40.7 μV for PBK-modified microelectrode and 26.0 ± 5.8 µV for PBK/PGO modified microelectrode, respectively. Meanwhile, as depicted in Figure 6(a-3,b-3,c-3), the averaged amplitude of the band-pass PEC noise dropped from 38.0 ± 4.9 µV to 28.0 ± 4.6 µV and submerged with the background noise accordingly. The results show that the PEC noise of the PI-based probes could be significantly reduced after PBK/PGO modification. This was because the band gap of PGO was larger than the photon energy emitted by the blue laser, which prevented most photons from being absorbed by PGO, thus weakening the PEC effect. In addition, the PEC noise had little influence on the high-frequency neuron action potential, but significant influence on the low-frequency local field potential.

In order to study the effect of silicon substrate on the photoelectric artifacts, the flexible PI-based probe was attached to a silicon substrate for the photoelectric artifact test. As shown in Figure 6(a-4,b-4,c-4), the amplitude of the photoelectric artifacts of the silicon-based probe were much higher than those of the PI-based probe. The increased amplitude of photoelectric artifacts originated from the PV effect of the silicon substrate. If the PEC noise is ignored, the averaged amplitude of the PV-induced artifact drops from 605.0 ± 62.7 μV of unmodified microelectrode to 247.0 ± 23.7 μV of PBK-modified microelectrode 176.0 ± 13.9 μV of PBK/PGO modified microelectrode, respectively, implying that the PV noise is also closely related to the electrode impedance. Therefore, the PV noise was a type of common mode noise, and it could be decreased by reducing the electrode impedance or be completely eliminated by replacing the silicon substrate with the polymer substrate. It is worth mentioning that the waveform recovery time of PEC noise was significantly longer than that of PV noise, which means that the PEC noise was more easily eliminated by bandpass filtering. As a result, the PEC noise had a smaller influence on neuron action potential than the PV noise.

## 3. Materials and Methods

### 3.1. Reagents and Apparatus

GO solution (1 mg/mL) was purchased from Suzhou TANFENG Graphene Technology Co., Ltd. (Suzhou, China). Phosphate buffered saline (PBS, pH 7.4) and PEG-6000 were purchased from Sinopharm Chemical Reagent Co., Ltd. (Shanghai, China). EDOT and chloroplatinic acid hexahydrate were purchased from Cool Chemical Technology (Beijing, China) Co., Ltd. Agar powder was purchased from Beijing Chembase Co., Ltd. (Beijing, China). The laser diodes (445 nm, 80 mW) were purchased from SINOSEMIC Co., Ltd. (Jinan, China). Anisotropic conductive films (ACF) were purchased from HITACHI, Ltd. (Tokyo, Japan). Scanning electron microscopy (SEM) analysis was performed using a high vacuum scanning electron microscope (ULTRA55, Zeiss, Germany). The electrochemical measurements and modifications were conducted with an electrochemical workstation (CHI660E, CH instrument). Noise signals were recorded using an RHD2000 Evaluation System (Intan Technology, LA, CA, USA).

### 3.2. Probe Design

**Figure 7:**
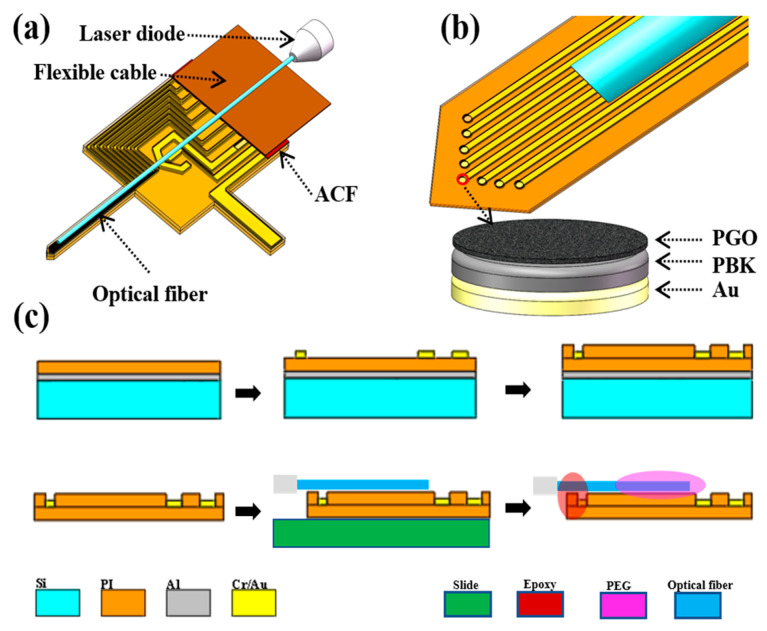
Schematic diagram of (**a**) assembled probe and (**b**) modified microelectrodes. (**c**) Fabrication processes of PI neural probe.

The structure of the integrated probe, depicted in Figure 7a, was composed of a flexible PI-based probe, an optical fiber, and a flexible conductive cable. The flexible neural probe was prepared by micromachining techniques, and the encapsulation material was polyimide. As shown in Figure 7b, the probe has a shank with a length of 5 mm, a width of 420 μm, and a thickness of 53 μm. A total of eight microelectrodes with a diameter of 25 μm and a pitch of 70 μm were arranged on the tip of the shank in the shape of an arrow. The microelectrodes were modified with PBK/PGO double layer to improve the sensitivity and resolution of the probe. The signal transmission between the flexible neural probe and the cable was realized by ACF based hot-press bonding [45]. An optical fiber with cladding/core diameter of 260/105 µm was attached to the surface of the flexible PI probe, and the distance between the recording microelectrodes and the optical fiber was about 0.2 mm. The flexible probe and the optical fiber were fixed together using polyethylene glycol (PEG) as adhesive. The PEG can degrade in tissue fluid after implantation, allowing the PI-based probe to regain its flexibility. Therefore, this design allows the probe to be rigid enough to pierce brain tissue during implantation and flexible enough to reduce mechanical mismatches after implantation.

### 3.3. Probe Fabrication

Figure 7c depicts the fabrication processes for the flexible neural probes used for photoelectric integration. Firstly, a double-side polished and oxidized P-type silicon wafer was chosen as the substrate. The thicknesses of the silicon layer and the oxide layer of the wafer were 500 µm and 0.5 µm, respectively. Then, a layer of 300 nm-thick aluminum was evaporated as a sacrificial layer on the wafer by an E-Beam Evaporation System. After that, a layer of 50 μm-thick PI tape (3M China Co., Ltd.) was attached to the wafer through a film laminator to form the lower insulating layer (step 1). Then, the Cr/Au layers (20/200 nm) were sputtered and patterned to form the microelectrode sites, conductive traces, and bonding pads (steps 2). After that, a layer of 3 μm-thick PI resin (PAA-1002, Changzhou ya’an new material Co. LTD, Changzhou, China) was spin-coated to form the top insulating layer. The specific curing steps of PI included heating on a hot plate at 80 °C for 20 min, 120 °C for 20 min, 150 °C for 30 min, 180 °C for 30 min, 200 °C for 20 min, 220 °C for 10 min, 250 °C for 10 min, and then cooling naturally. Then, a layer of 100 nm thick Cu was sputtered and patterned to serve as hard mask. Next, the exposed Cu was removed by wet etching, and the microelectrode sites and bonding pads were exposed by reactive ion etching (RIE) (step 3). Finally, the wafer was put into HCl aqueous solution (10%) to release the neural probes from the substrate (step 4).

### 3.4. Optoelectronic Integration

To achieve photoelectric integration, the flexible probe was fixed on a glass slide by Van der Waals’ force firstly. A multimode glass fiber (0.22 NA) with a core/cladding diameter of 105/260 μm was used as the optical waveguide for coupling with a TO-56 packaged laser diode. The fiber was cut to a specific length, and its output end was polished with sandpapers of different roughness. After remove the cladding layer of the input end of the fiber, the core fiber was inserted into a ceramic ferrule with an inner diameter of 0.125 mm and outer diameter of 2.5 mm. Then, the fiber was fixed with the ceramic ferrule by UV-curing adhesive. For integration, the flexible probe was placed on the stage of an ultrasonic wire-bonding machine, and the temperature was raised to 100 °C. The fiber was gently placed on the probe surface, and the position of the fiber was adjusted through the Gaiser wire bonder under microscope to align it with the recording microelectrodes (step 5). After the alignment, the fiber was coated with PEG via an electric soldering iron, and then the temperature of the stage was reduced to room temperature to achieve temporary fixation of the optical fiber. To avoid disconnection between the fiber and the neural probe, the end of the fiber and the base of the probe were fixed together with an epoxy resin adhesive (step 6). As the TO-56 packaged LD (Beijing Lightsensing Technologies Ltd., Beijing, China) also had a multimode glass fiber with core diameter of 105 μm, and its output end was connected to the same ceramic ferrule, the optical coupling could be easily realized by a ceramic mating sleeve with an inner diameter of 2.5 mm.

### 3.5. Electrochemical Modification

To reduce the photoelectric artifacts and improve the electrochemical performance of the flexible PI-based probe, the eight microelectrodes were modified with PBK and PGO, successively. In detail, PBK was electroplated by applying repetitive current pulses (duty ratio of 5 ms: 495 ms, peak current density of 4.5 A/cm^2^, cycles of 200) in chloroplatinic acid solution (3% chloroplatinic acid and 0.01% lead acetate in deionized water). For PGO deposition, GO aqueous solution (1 mg/mL) was ultrasonicated for 30 min to disperse it evenly. Then, 0.01 M ethylenedioxythiophene (EDOT) was added, and the solution was stirred for 1 h to obtain the electrolyte. The PGO was electrodeposited on PBK modified electrodes by applying repetitive current pulses (duty ratio of 5 ms: 495 ms, peak current density of 0.83 A/cm^2^, cycles of 100). The current pulses were generated by an electrochemical workstation (CHI660E, CH instrument, Shanghai, China) with a Pt wire as reference and counter electrodes.

### 3.6. Bench Noise Measurement

The flexible probe was fixed on a glass/silicon slide with PI tape, and the microelectrodes were inserted into PBS solution to measure optical stimulation noise in a foil shielded box. The “Multi-Current Steps” technology of the electrochemical workstation was applied to provide the driven current to the LD. The signal recording system included an RHD USB interface board, an RHD SPI interface cable, and an RHD 32-channel headstage (Intan Technology, LA, CA, USA). In order to realize signal recording, the platinum wire was selected as ground and reference electrode, with the eight microelectrodes of the probe as recording electrodes. The signal was band-pass filtered from 250 to 8000 Hz for high frequency analysis. The 445 nm blue laser pulses with a frequency of 10 Hz and a duration of 5 ms were applied to induce the photoelectric artifacts. The bias current of the LD was 10 mA, and the drive current was 100/200 mA. The corresponding optical powers of the output end of the fiber in air were 278.37 mW/mm^2^ and 641.79 mW/mm^2^, respectively. The maximum voltage offset at the moment the laser pulse was turned on was defined as the amplitude of the photoelectric artifact.

## 4. Conclusions

In conclusion, a flexible polyimide-based probe with PBK/PGO modified microelectrodes was developed to improve electrochemical performance and reduce optical stimulation artifacts. The CV and EIS results showed that the PBK/PGO modified probe had the best electrochemical performance compare to unmodified and PBK modified probes. In addition, the bench noise recordings revealed that the amplitude of PV noise was mainly related to the electrode impedance, while the amplitude of PEC noise mainly depended on the energy band structure of the electrode material. To be more specific, the PBK layer mainly suppressed PV noise by decreasing the impedance of the microelectrodes, while the PGO layer mainly suppressed PEC noise by reducing the number of residual photo-induced carriers. In comparison, PV noise had greater influence on neuron action potential than PEC noise. Fortunately, the high-amplitude PV noise can be completely eliminated by replacing the silicon substrate with the polymer substrate. Therefore, this electrochemically modified flexible PI-based probe has important value for high-resolution optogenetics applications.

## Figures and Tables

**Table 1:** The dictionary of abbreviations.

Item	Paraphrase
PEG	polyethylene glycol
PEDOT	poly(3,4-ethylene-dioxythiophene)
PSS	polystyrene sulfonic acid
GO	graphene oxide
PBK	Platinum-Black
PGO	graphene oxide doped poly(3,4-ethylene-dioxythiophene)
PEC	photoelectrochemical
PV	photovoltaic
LED	light emitting diode
LD	Laser diode
EMI	electromagnetic interference
SEM	scanning electron microscopy
SCE	saturated calomel electrode
CV	cyclic voltammograms
EIS	electrochemical impedance spectra
CSC	charge storage capacity
PBS	phosphate buffered saline
ACF	anisotropic conductive films
SNR	signal-to-noise ratio

**Table 2:** Comparison of the reported methods for suppression of optical stimulation-induced noise.

References	Noises	Substrate	Methods	Total Noise Amplitude	Irradiance Power
[40]	PEC	Silicon	PEDOT:PSS/graphene modification	40 µV(positive)	30 mW/mm^2^
[41]	PEC	Silicon	PBK/PSS modification	250 µV (negative)	38.2 mW/mm^2^
This work	PEC	PI	PBK/PGO modification	50 µV (positive)	641.79 mW/mm^2^
[23]	PV	Silicon	heavily boron-doping	50 µV (peak-peak)	50 mW/mm^2^
[41]	PV	Silicon	PBK/PSS modification	250 µV (negative)	38.2 mW/mm^2^
This work	PV	Silicon	PBK/PGO modification	176 μV (negative)	278.37 mW/mm^2^
[21]	EMI	Silicon	Grounded brass shield	100 µV (positive)	2142 mW/mm^2^
[23]	EMI	Silicon	Metal shielding layer	50 µV (peak-peak)	50 mW/mm^2^

**Table 3:** The numerical fitting results of equivalent circuit components of PBK/PGO coated microelectrodes before and after stability tests.

Item	C_dl_ (F)	R_ct_ (Ω)	Y^0^ (S·s^0.5^)	C_d_ (F)	χ^2^
PBK/PGO ^1^	1.551 × 10^−7^	4.216 × 10^3^	1.490 × 10^−5^	5.202 × 10^−10^	1.210 × 10^−2^
PBK/PGO ^1^ after CV scanning	1.019 × 10^−7^ ↓	4.745 × 10^3^ ↑	1.692 × 10^−6^ ↓	3.604 × 10^−10^ ↓	2.405 × 10^−2^ ↑
PBK/PGO ^2^	9.729 × 10^−8^	1.532 × 10^4^	4.389 × 10−^6^	1.321 × 10^−10^	3.305 × 10^−3^
PBK/PGO ^2^ after sonication	1.100 × 10^−7^ ↑	2.192 × 10^4^ ↑	1.198 × 10^−6^ ↓	3.360 × 10^−10^ ↑	2.304 × 10^−2^ ↑

^1^ Sample a; ^2^ Sample b.
